# Active Surveillance and Farm-Level Risk Evaluation of African Swine Fever in Southern Nigeria

**DOI:** 10.3390/pathogens14090934

**Published:** 2025-09-16

**Authors:** Alhaji S. Olono, Olusola A. Ogunsanya, Ayotunde E. Sijuwola, Femi M. Saibu, Oluwatobi Adedokun, Akeemat O. Ayinla, John Fadele, Harouna Soumare, Eugenie Y. Tchokote, John O. Abiola, Bonto Faburay, Corrie Brown, Christian T. Happi, Anise N. Happi

**Affiliations:** 1 The Institute of Genomics and Global Health, Redeemer’s University, Ede 232102, Osun State, Nigeria; olono9902@run.edu.ng (A.S.O.); ogunsanyao@run.edu.ng (O.A.O.); sijuwolaa@run.edu.ng (A.E.S.); saibum@run.edu.ng (F.M.S.); adedokunolu@run.edu.ng (O.A.); ayinlaa@run.edu.ng (A.O.A.); fadele15895@run.edu.ng (J.F.); soumareh@run.edu.ng (H.S.); happic@run.edu.ng (C.T.H.); 2 Department of Biological Sciences, Redeemer’s University, Ede 232102, Osun State, Nigeria; 3 Department of Veterinary Medicine, College of Veterinary Medicine, Michael Okpara University of Agriculture, Umudike 440101, Abia State, Nigeria; tchokote.eugenie@mouau.edu.ng; 4 Department of Veterinary Medicine, University of Ibadan, Ibadan 200284, Oyo State, Nigeria; jo.abiola@mail.ui.edu.ng; 5 Foreign Animal Disease Diagnostic Laboratory, National Bio and Agro-Defense Facility, Animal and Plant Health Inspection Service, U.S. Department of Agriculture, Manhattan, KS 66502, USA; bonto.faburay@usda.gov; 6 LifeStock International, Athens, GA 30606, USA; corrie@lifestock.org

**Keywords:** biosecurity, genotype, African swine fever, surveillance, seroprevalence, Nigeria, ticks

## Abstract

Pig farms in Southern Nigeria face recurrent threats from enzootic viral infections, yet active surveillance remains limited. This study implemented an active surveillance approach targeting African swine fever virus (ASFV) to assess its circulation across four states. We sampled 40 pig farms and two abattoirs, collecting swine blood and ticks for molecular and serological analysis. Sampling was conducted during both African swine fever (ASF) outbreak (*n* = 27 pigs) and non-outbreak (*n* = 204 pigs) periods, resulting in 231 samples tested for ASFV DNA. Additionally, 46 plasma samples from the non-outbreak period were screened for ASFV-specific antibodies. ASFV was detected in all pigs sampled during outbreak periods (27/27; 100%), whereas none of the pigs sampled during non-outbreak periods tested positive (0/204). Of the 27 positives, sequencing generated 7 genomes characterized as genotype II. Serological testing of the 46 plasma samples from non-outbreak pigs revealed no ASFV seropositivity. All tick samples were negative for ASFV DNA. Farm-level risk evaluations revealed widespread biosecurity lapses, suggesting these deficiencies as potential drivers of ASFV circulation. These findings underscore the urgent need to strengthen farm-level biosecurity and demonstrate the pivotal role of active surveillance and genome sequencing in detecting and characterizing ASF outbreaks.

## 1. Introduction

African Swine Fever (ASF) is a highly contagious viral hemorrhagic disease affecting domestic pigs with severe negative implications for food security and developing economies. Since its introduction into Nigeria over two decades ago, ASF has caused near-annual epizootic-scale outbreaks with devastating impacts on smallholder pig farmers whose livelihoods depend heavily on swine production [1,2,3,4]. These resurgent outbreaks have raised pressing questions about the virus, particularly the genotypes in circulation and the factor(s) fueling its persistence.

Until recently, genotype I had been the dominant variant in Nigeria. However, the recent identification of genotype II marked a turning point, suggesting a potential shift in the epidemiology of ASF within the country [5]. This discovery underscores the need for active surveillance to determine whether the current wave of infections is driven by genotype II, genotype I, both, or even an entirely new genotype, prompting this study to sample pigs during outbreak periods. Knowledge of the outbreak-causing genotype(s) is crucial for tracking the transboundary spread and evaluating implications for vaccine development and regional control strategies.

Furthermore, to unravel some of the factors driving the resurgence, we assessed pigs, ticks, and farm behaviors during non-outbreak periods. During this phase, evaluating molecular and seroprevalence in pigs offers valuable insights into subclinical circulation and population-level immunity. Serological data can reveal past exposure in apparently healthy pigs, helping to identify silent transmission chains and guide targeted interventions. The sampling of ticks found in close association with the pigs will also help us understand the role this arthropod plays in the persistence of African swine fever virus (ASFV) within the study areas.

Finally, evaluating farm-level practices during non-outbreak periods allows us to identify behaviors that may contribute to outbreak resurgence and shape disease dynamics. When combined with molecular and serological data, these observations provide valuable insights to guide targeted interventions and inform future control strategies.

## 2. Materials and Methods

### 2.1. Ethical Approval

The study was conducted in accordance with national and institutional guidelines, with ethical approval obtained from NHREC (NHREC/01/01/2007) and AUCC (AEC/02/123/22). All sampling procedures adhered to animal welfare standards and were performed exclusively by licensed veterinarians. Prior to blood sample collection, farmers were informed of the survey’s objectives and rationale. Participation was entirely voluntary, with individuals free to withdraw at any point.

### 2.2. Study Area

**Figure 1:**
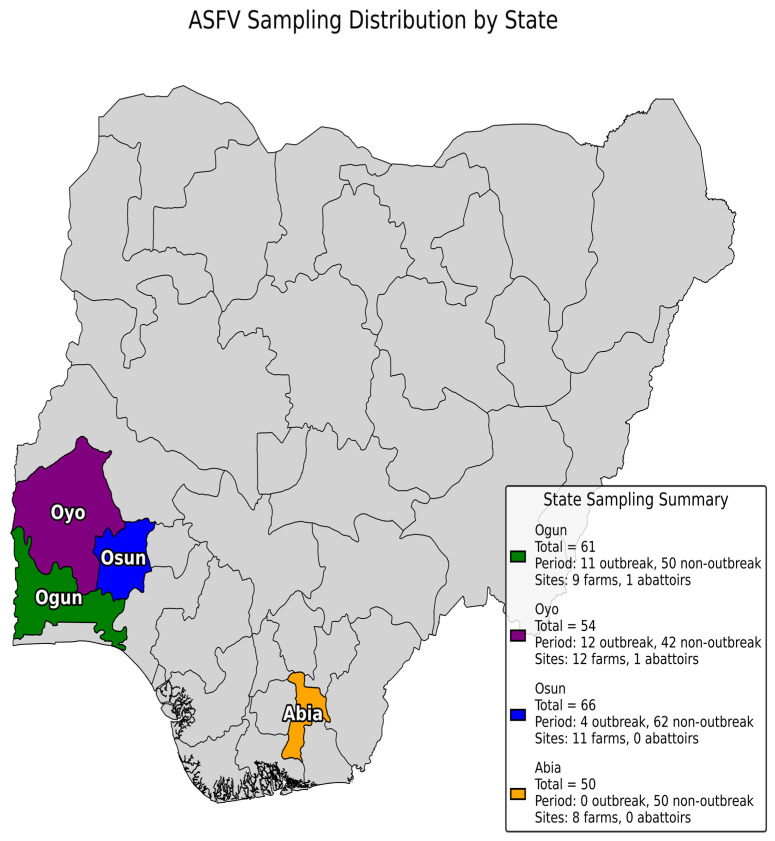
Map of Nigeria highlighting the four states where sampling was performed. A brief summary of the number of samples collected from each state and the sampling period in which they were collected, and the number of sites sampled is also displayed.

This study was carried out in four states in Southern Nigeria. In Ogun and Oyo States, we visited both pig farms and abattoirs. The abattoirs were mixed-species facilities, each with a dedicated section for pig slaughter. In Osun and Abia States, the investigation focused only on pig farms (Figure 1).

### 2.3. Study Design and Field Data Collection

A cross-sectional descriptive study was conducted between September 2022 and August 2023. We visited 40 pig farms and two abattoirs and collected 231 whole blood samples for ASFV analysis—a number determined by the resources and logistical constraints of active field surveillance. Sampling took place during two outbreak windows (September–October 2022 and July–August 2023) and a non-outbreak period (January–July 2023), with the distribution of samples by site and period detailed in Table S1.

During non-outbreak periods, sites were chosen at random, and visits combined blood collection with tick sampling and farm data collection. Data collection comprised two main components: (1) standardized questionnaires administered to farm personnel, and (2) farm-level risk assessments conducted by the research team. Both approaches focused on risk factors for ASFV. In contrast, during the outbreak windows, farms with active ASF cases were chosen based on prior outbreak reports, and visits focused exclusively on blood sample collection; no arthropod sampling, questionnaires, or risk assessments were performed.

The risk assessment process was conducted in two stages. First, we identified and extracted relevant risk factors for ASF from existing literature, organizing them into two broad categories: environmental and managemental factors [6,7,8,9,10,11]. In the second stage, each farm was evaluated for the presence of these factors. For environmental risk, three specific factors were assessed (Table 1). Farms that exhibited all three were classified as high risk in this category. Those with two out of three were considered medium risk, and farms with only one factor present were categorized as low risk. For managemental risk, ten factors were assessed (Table 1). Farms that demonstrated seven or more of these were classified as high risk, those with four to six were considered medium risk, and farms with three or fewer were categorized as low risk.

### 2.4. Sampling, Processing, and Storage

#### 2.4.1. Pig Blood

From each pig, 5 mL of whole blood was collected via cranial vena cava venipuncture using a sterile needle and syringe. A 1.5 mL aliquot was preserved in DNA/RNA Shield™ (Zymo Research; Irvine, CA, USA) for ASFV nucleic acid testing. The remaining 3.5 mL was centrifuged at 3000 rpm for 10 min to obtain plasma for serology. All samples were stored at −20 °C until analysis.

#### 2.4.2. Tick Sampling

Hard ticks (not speciated and unsexed) were collected from pigs and their immediate environment from a total of four farms—two each in Ogun and Osun States. A total of fourteen ticks were obtained. After collection, each tick was bisected longitudinally using a binocular dissecting microscope (Olympus; Tokyo, Japan). Halves were then pooled in groups (a maximum of five halves per pool), yielding four pools (a pool per farm). All pools were preserved in DNA/RNA Shield™ and stored at −20 °C until further processing.

### 2.5. Nucleic Acid Detection in Pigs and Ticks

Total DNA from 231 blood samples and from the four tick pools was extracted using the QIAamp Viral DNA Extraction Kit (Qiagen; Hilden, Germany). Tick pools were homogenized with glass beads prior to DNA extraction. PCR targeting p72 and p54 (for samples inconclusive by p72) genes was carried out using a 25 µL reaction mix: 22 µL of lyophilized PuReTaq™ Ready-To-Go™ PCR mix (Cytiva; Marlborough, MA, USA), 0.25 µM of each primer, and 3 µL of DNA extract, with all results benchmarked against a positive control [12,13].

### 2.6. Antibody Detection in Pigs

ASFV-specific antibodies were screened in 46 randomly selected plasma samples (from the non-outbreak period) using the Elabscience® indirect ELISA kit (E-AD-E106) (Elabscience Biotechnology; Wuhan, China) coated with p30 antigens (the details of the sample distribution are provided in Table S2). Per manufacturer’s instructions, samples were classified as positive (S/P ≥ 50%), negative (S/P < 40%), or invalid (S/P 40–50%).

### 2.7. Whole-Genome Sequencing

Selected ASFV-positive samples (extracted DNA) were quantified using the Qubit™ High Sensitivity DNA Assay (Invitrogen; Waltham, MA, USA). Host DNA was removed using the NEBNext^®^ Microbiome DNA Enrichment Kit (New England Biolabs; Ipswich, MA, USA), after which libraries were prepared and sequenced on the Oxford Nanopore GridION (Oxford Nanopore Technologies; Oxford, UK) platform using MinKNOW™ software (v23.04.6).

### 2.8. Statistical and Bioinformatic Analysis

#### 2.8.1. Statistical Analysis

Descriptive analyses using Python v3.11 (Pandas library v2.2.1) were employed to summarize farm practices and risk scores obtained from questionnaires and farm assessments. Matplotlib (v3.10.3) was used for visualizations.

#### 2.8.2. Reference-Based Genome Assembly and Genotype Assignment

High-quality reads (Q score ≥ 9) were aligned to 17 ASFV reference genomes using minimap2 (v2.30). The reference genome with the highest coverage and percentage identity was used for assigning the genotypes. Only samples with an average read depth of 20× were considered adequate for genotype assignment.

## 3. Results

### 3.1. PCR, Serology, and Genotyping Results

Out of 231 pig blood samples collected across four Nigerian states, PCR screening using p72 primers confirmed ASFV in 27 pigs (the details of the positivity across sampling periods and sites are provided in Table S1). All positives were detected during the outbreak sampling period, representing a 100% positivity rate among samples collected during that time. Notably, 0 out of the 204 samples tested from the non-outbreak periods were positive. Additionally, all four pools of ticks (representing 14 ticks, all hard ticks) tested negative by PCR. All 46 plasma samples screened for ASFV antibodies were seronegative.

Reference-based genome assembly was carried out on 13 ASF-positive samples. Seven complete genome assemblies were generated. These genomes were from two farms out of the three sampled in Oyo State (Table S1), with each genome showing 96.5–96.8% genome coverage. Their read depths range from 127 to 435 reads per locus. All assemblies aligned best to the Georgia genotype II reference strain (NC_044959.2).

### 3.2. Demographics

**Figure 2:**
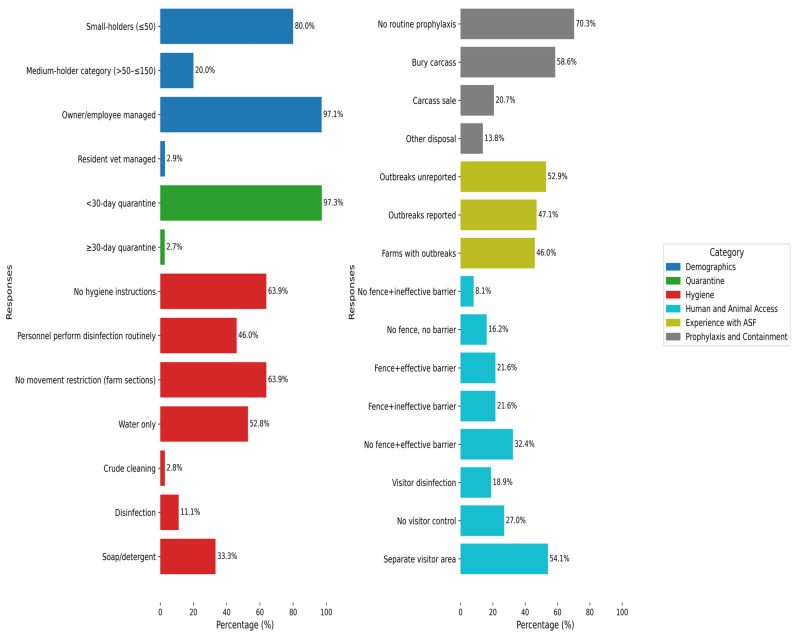
Responses to the ASF questionnaire by category. The horizontal bar chart illustrates the proportion of farms that responded to the questions. Percentage values are displayed at the tip of each bar, and response categories are color-coded as indicated in the legend.

Among farms providing demographic information (holding capacity, location, and manager; *n* = 35), small-holder units (≤50 pigs), peri-urban (54.3%), and rural (25.7%) settings dominated the sampling, with an overwhelming majority of farms managed by owners/employees (Figure 2). Farm distribution by state (*n* = 37) was relatively balanced: Oyo and Osun each contributed 27.0%, Ogun 24.3%, and Abia 21.6%. Ogun and Oyo States each accounted for 50% of the sampled abattoirs (*n* = 2).

### 3.3. Farm Risk Evaluation to ASFV Infection Using Questionnaire Findings

#### 3.3.1. Sourcing and Quarantine (*N* = 37)

Almost two-thirds of farms (64.7%) sourced pigs from distant locations {i.e., geographical locations considered far from the farm; pigs are transported long distances (≈60–80 km) in order to reach the farm}. In addition, using the farm’s own breeding stock (i.e., retaining pigs born on-site for restocking) was also a common practice (62.2%). Other approaches included sourcing pigs from nearby farms (51.6%) and importing pigs from outside Nigeria (5.4%). Although most farms claimed to quarantine new arrivals, in reality, they fell short of recommended practice, with most quarantining pigs for 7–14 days (<30 days) (Figure 2).

#### 3.3.2. Hygiene and Movement Control

Based on general hygiene protocols and personnel hygiene (*n* = 36), nearly two-thirds of farms lacked documented hygiene protocols {14 (60.9%) of these had a previous history of ASF outbreak} and relied exclusively on water for cleaning waste and blood-contaminated areas. Fewer than half implemented personnel disinfection or restricted movement between farm sections (Figure 2). A small proportion of farms (2.8%) reported using crude cleaning methods, such as manually removing wastes from pens without washing or disinfecting, or wiping off liquid waste and blood stains with only cloths (Figure 2). With regard to equipment management (*n* = 37), a majority (91.9%) preferred purchasing tools over borrowing or lending. Cleaning and disinfection practices of these tools varied but were relatively evenly distributed: 12 farms (32.4%) each reported cleaning either after use, on occasion, or both before and after use. Five farms (13.5%) did not use any disinfectant (*n* = 37). IZAL, a phenol-based disinfectant, was the most commonly reported product (24.3%), followed by combinations of formalin and hypochlorite (5.4%), along with several others.

#### 3.3.3. Human and Animal Access (*N* = 37)

Structural biosecurity measures were generally insufficient, with many farms lacking effective fencing, permitting contact between pigs and wild or stray animals and birds. Also, many farms implemented inadequate visitor control protocols (Figure 2). Concerning the introduction of pigs from external sources, 73% of farms fully restricted such entries. Among the minority that allowed them, amongst other reasons, the primary reason cited was stud service (21.6%).

#### 3.3.4. Experience with ASF Outbreaks (*N* = 37)

Approximately 46% of farms reported having experienced an outbreak; however, only about half of these incidents were communicated to the authorities (Figure 2). Among the unreported cases, the most common reason cited was a perceived lack of support (77.8%), followed by fear of closure (11.1%), while the remaining 11.1% offered no specific justification.

#### 3.3.5. Prophylaxis and Carcass Management

For prophylactic practices (*n* = 37), routine vaccination and deworming practices were largely absent across farms (Figure 2). Additionally, most farms (56.7%) did not consult veterinarians during pig procurement, with only one farm (2.7%) engaging veterinary services exclusively at the point of purchase.

Carcass disposal practices (*n* = 29) varied among farms, ranging from on-site burial (on or near the farmland) to the sale of carcasses (Figure 2). Additional methods included disposal in bushes (2 farms; 6.9%), rivers (1 farm; 3.5%), and dunghills (1 farm; 3.5%). Two farms (6.9%) reported no pig mortality at the time of the survey.

### 3.4. Farm Risk Evaluation to ASFV Infection Using Farm Assessment

**Figure 3:**
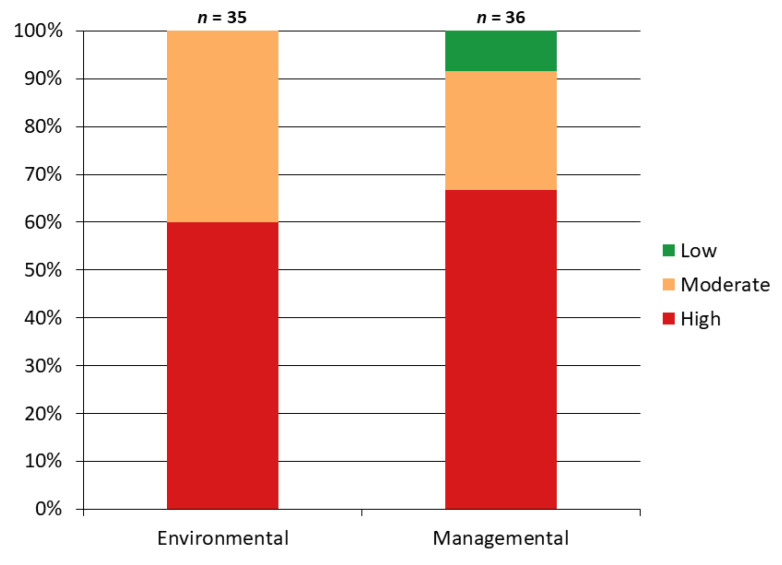
Distribution of African Swine Fever risk levels across surveyed pig farms. Stacked bar charts show that most farms have a high risk across both considered categories.

**Figure 4:**
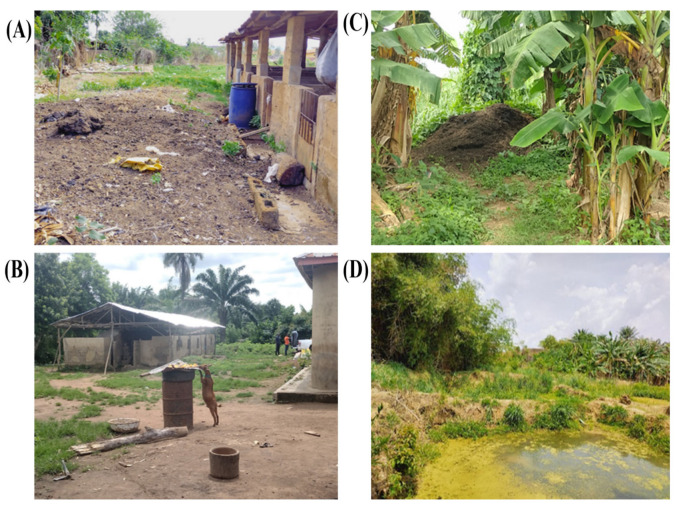
Examples of biosecurity breaches observed on surveyed farms. (**A**) Improper disposal of fecal waste, with litter scattered around the perimeter of the pen; (**B**) unrestricted farm access, as evidenced by a stray goat accessing feed materials; (**C**) fecal waste stored in close proximity to animal housing; (**D**) use of untreated surface water, with multiple containers dipped directly into the source.

The majority of the farms had a high environmental and managemental risk of ASF (Figure 3). Also, Figure 4 captures some visual examples of biosecurity breaches observed during the farm assessment.

## 4. Discussion

This study demonstrates that ASFV positivity was high in samples collected during outbreak periods, with genotype II exclusively detected among genotyped cases. The virus and its antibodies were absent in pigs during non-outbreak phases. ASFV DNA was not detected in hard ticks closely associated with pigs. The study thus suggests that widespread farm-level biosecurity lapses, rather than subclinical transmission or tick reservoirs, are likely drivers of pathogen transmission.

The identification of ASFV genotype II in positive samples during the outbreak period corroborates the growing nature of this genotype in Nigeria [5], aligning with global epidemiological patterns. This finding also corroborates previous reports that designate genotype II as the primary genotype responsible for the current global pig pandemic [14,15]. In the areas sampled and the viruses genotyped, it appears that ASFV genotype II may be replacing ASFV genotype I. The knowledge of these outbreak-causing genotype(s) is crucial for tracking transboundary spread (genotype II is currently the globally implicated cause of outbreaks [14,15]) and for evaluating implications on vaccine development and regional control strategies. However, it is important to emphasize that only 7 of the 27 positive samples collected during the outbreak period were successfully genotyped. Consequently, the identified genotype II sequences may not fully capture the diversity of ASFV genotypes circulating at that time. In addition to molecular detection, serological screening of 46 plasma samples collected during non-outbreak periods revealed no detectable antibodies. This suggests that pigs in the study population may have had little to no prior exposure to ASFV. Our observations are consistent with a recent serological survey conducted in Nigeria, which, although performed in a different region, also reported very low antibody titers among farmed pigs [16]. Earlier studies in our study area reported higher farm-level seroprevalence; however, these studies are over a decade old, and their findings are likely outdated given the relatively short lifespan of domestic pigs in farm settings [8,17]. The findings from our study, therefore, provide a more current perspective and are particularly significant, as they may indicate limited or absence of subclinical circulation or silent transmission chains in the study region. Alternatively, they could also reflect effective culling practices. More critically, however, the lack of seropositivity highlights a potential vulnerability in herd immunity, implying that these pigs may be immunologically naive and therefore highly susceptible to future outbreaks (even by those caused by the same circulating genotype(s) within the study area. This study also suggests that the recurrent nature of ASF appears largely shaped by structural and human-inclined risk factors, particularly inadequate infrastructure, suboptimal management practices, and economically motivated behaviors rather than subclinical transmission, chronic carriers, or tick vectors. A key structural weakness observed was the predominance of smallholder operations—most farms housed ≤ 50 pigs and were managed by owners or staff with limited veterinary oversight. This pattern mirrors findings from other ASF-endemic regions, where the subsistence nature of pig farming often results in weak or poorly enforced biosecurity frameworks [18,19,20,21], fostering an environment highly conducive to ASFV transmission. Clearly, to control ASF spread within southern Nigeria, awareness regarding proper management practices and improved biosecurity measures is needed. Specifically, behaviors such as observing inadequate quarantine practices as well as hygiene deficiencies, due to their pervasive nature within the study population, are likely to have facilitated the spread and resurgence of ASFV. Also, disease containment practices following pig mortalities remained inadequate. Farmers frequently resorted to improper carcass disposal—such as emergency sales, on-site burials, and discarding remains in bushes or waterways—thereby increasing the risk of environmental transmission. These practices are likely to be influenced by limited awareness of proper biosecurity protocols and economic constraints.

Although Ornithodoros soft ticks are well-documented as the primary vectors of ASFV [22,23], our field surveys, comprising thorough inspections of pig pens and adjacent environments, yielded only hard ticks, all of which tested negative for ASFV DNA. This finding aligns with current knowledge, as evidence supporting the role of hard ticks as vectors of ASFV remains limited [24]. The lack of soft tick capture in this study is not surprising, as it aligns with previous studies reporting limited presence of the well-known ASFV-transmitting soft tick in West Africa [25,26]. Another species of soft ticks, which are widespread in West Africa and could be important in the persistence of the pathogen [27,28], are mostly associated with rodent burrows, which were not sampled in this study. It is therefore safe to assume that ASFV persistence in the study region is predominantly driven by direct pig-to-pig transmission (i.e., the domestic) cycle rather than by a tick–warthog (sylvatic) cycle. However, the small number of ticks collected and their limited geographic distribution reduce the representativeness of the sample and constrain the strength of conclusions regarding hard tick involvement in ASFV transmission within the study area.

## 5. Conclusions

This study highlights the necessity of proactive surveillance in generating critical data for understanding disease epidemiology and informing targeted intervention strategies. Multifactorial biosecurity lapses appear to be the primary drivers of the recurrent epizootic-scale outbreaks in Southern Nigeria. Given the potential vulnerability in herd immunity, strengthening biosecurity measures, implementing policy interventions that provide financial or material support for farm infrastructure and compensation for losses, incentivizing outbreak reporting, and enhancing farmer education on effective disease prevention and control are essential steps toward mitigating recurrent outbreaks.

## Figures and Tables

**Table 1:** Criteria for Classifying Farms by Environmental and Managemental Risk in African Swine Fever (ASF) Farm-level Risk Assessment.

Category	Factors Considered for Classification
Environmental	High pig farm density (i.e., ≈2 km radius around sampled farm) *, lack of perimeter fencing, farm located in a valley/flood-prone site.
Managemental	Extensive production system, unrestricted visitors’ access, unrestricted workers’ access, absence of vehicular/foot dips, poor feeding and feed storage practices, unpurified surface water, poor housing and hygiene, presence of onsite slaughter, multiple/no veterinary presence *, the use of the same injection for multiple animals.

* Information was retrieved by both physical assessment and discussion with the farmer.

## Data Availability

The original contributions presented in this study are included in the article/Supplementary Materials. Further inquiries can be directed to the corresponding author(s).

## Supplementary Materials

The following supporting information can be downloaded at https://www.mdpi.com/article/10.3390/pathogens14090934/s1, Table S1: Sample distribution, ASFV PCR positivity, and genome assembly status across the four sampled Nigerian states, by sampling period and site. Table S2: Summary of Farm and Pig Sampling, Testing Coverage, and African Swine Fever Virus (ASFV) Seropositivity Across Four Nigerian States.

## Abbreviations

The following abbreviations are used in this manuscript:

ASFV	African swine fever virus
ASF	African swine fever
NHREC	National Health Research Ethics Committee
AUCC	Animal Use and Care Committee
RT-qPCR	Reverse transcription quantitative polymerase chain reaction
PCR	Polymerase chain reaction
DNA	Deoxyribonucleic acid
ELISA	Enzyme-linked immunosorbent assay
OD	Optical density
