# Supplementary material for: Active Surveillance and Farm-Level Risk Evaluation of African Swine Fever in Southern Nigeria
Source: Pathogens. 2025 Sep 16;14(9):934. doi: 10.3390/pathogens14090934 (PMC12472251; doi:10.3390/pathogens14090934)
Supplement: Supplementary file 1 [file pathogens-14-00934-s001.zip › Table S1.pdf]

Table S1. Sample distribution, ASFV PCR positivity, and genome assembly status across the four sampled Nigerian states, by sampling period and site.

State	Sampling Sites (Farms / Abattoirs)	Total Samples	Outbreak Period (n)	Non-Outbreak Period (n)	Farm Samples n (%)	Abattoir Samples n (%)	Farm-level Positivity n (%)	Abattoir-level Positivity n (%)	Genome Assembly Status
Ogun	9 / 1	61	11	50	50 (82.0%)	*11 (18.0%)	0 (0%)	*11 (100%)	None
Oyo	12 / 1<br><br>*3 Farms	54	12	42	52 (96.3%)<br><br>$42<br>*10	*2 (3.7%)	* 10 (100%)<br><br>*Farm 1: 1<br>*Farm 2: 3<br>*Farm 3: 6	*2 (100%)	*7 genomes;<br><br>*Farm 2: 1/3<br>*Farm 3: 6/6
Osun	11 / 0<br><br>*1 Farm	66	4	62	66 (100%)	0 (0%)	*4 (100%)	-	None
Abia	8 / 0	50	0	50	50 (100%)	0 (0%)	0 (0%)	-	-
**Total**	**40 / 2**	**231**	**27**	**204**	**218 (94.4%)**	***13 (5.6%)**	***14 (100%)**	***13 (100%)**	***7 genomes**

***= Associated with outbreak period, $ = Associated with non-outbreak period.**
