# Supplementary material for: Active Surveillance and Farm-Level Risk Evaluation of African Swine Fever in Southern Nigeria
Source: Pathogens. 2025 Sep 16;14(9):934. doi: 10.3390/pathogens14090934 (PMC12472251; doi:10.3390/pathogens14090934)
Supplement: Supplementary file 1 [file pathogens-14-00934-s001.zip › Table S2.pdf]

Table S2. Summary of Farm and Pig Sampling, Testing Coverage, and African Swine Fever Virus (ASFV) Seropositivity Across Four Nigerian States

State	Farms Sampled	Farms Tested n (%)	Total Samples Collected	Samples Tested for ASF-Ab n (%)
Ogun	9	3 (33.3)	50	18 (36)
Oyo	12	5 (41.7)	42	6 (14)
Osun	11	3 (27.3)	62	4 (6.5)
Abia	8	5 (62.5)	50	18 (26)
**Total**	**40**	**16 (40%)**	**204**	**46 (22.5)**

Ab= Antibodies.
